# 6-Methyl­pyridin-2-amine

**DOI:** 10.1107/S1600536812047800

**Published:** 2012-11-28

**Authors:** Sergiu Draguta, Victor N. Khrustalev, Bhupinder Sandhu, Mikhail Yu. Antipin, Tatiana V. Timofeeva

**Affiliations:** a D. Ghitu Institute of Electronic Engineering and Nanotechnologies, 3/3 Academy str., MD-2028, Chisinau, Republic of Moldova; b X-Ray Structural Centre, A.N. Nesmeyanov Institute of Organoelement Compounds, Russian Academy of Sciences, 28 Vavilov St, B-334, Moscow 119991, Russian Federation; c Department of Chemistry & Biology, New Mexico Highlands University, 803 University Avenue, Las Vegas, NM 87701, USA; d Department of Chemistry & Biology, New Mexico Highlands University, 803 University Avenue, Las mVegas, NM 87701, USA

## Abstract

In the title mol­ecule, C_6_H_8_N_2_, the endocyclic angles are in the range 118.43 (9)–122.65 (10)°. The mol­ecular skeleton is planar (r.m.s. deviation = 0.007 Å). One of the two amino H atoms is involved in an N—H⋯N hydrogen bond, forming an inversion dimer, while the other amino H atom participates in N—H⋯π inter­actions between the dimers, forming layers parallel to (100).

## Related literature

For general background to the design of chiral or acentric co-crystals, see: Jacques *et al.* (1981 ▶); Miyata (1991 ▶); Scheiner (1997 ▶). For related compounds, see: Büyükgüngör & Odabaşoğlu (2006 ▶); Chtioui & Jouini (2006 ▶); Ni *et al.* (2007 ▶); Dai *et al.* (2011 ▶); Waddell *et al.* (2011 ▶).
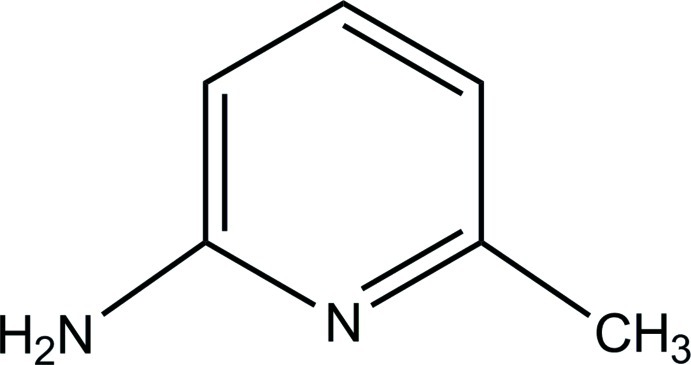


## Experimental

### 

#### Crystal data

C_6_H_8_N_2_
*M* *_r_* = 108.14
Monoclinic, 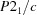

*a* = 9.1006 (11) Å
*b* = 6.2458 (8) Å
*c* = 10.5598 (13) Å
β = 100.952 (2)°
*V* = 589.29 (13) Å^3^
*Z* = 4
Mo *K*α radiation
μ = 0.08 mm^−1^
*T* = 296 K
0.30 × 0.25 × 0.20 mm

#### Data collection

Bruker APEXII CCD diffractometer
Absorption correction: multi-scan (*SADABS*; Sheldrick, 2003 ▶) *T*_min_ = 0.977, *T*_max_ = 0.985
5852 measured reflections
1420 independent reflections
1196 reflections with *I* > 2σ(*I*)
*R*_int_ = 0.030

#### Refinement

*R*[*F* ^2^ > 2σ(*F* ^2^)] = 0.043
*wR*(*F* ^2^) = 0.130
*S* = 1.00
1420 reflections
82 parameters
H atoms treated by a mixture of independent and constrained refinement
Δρ_max_ = 0.32 e Å^−3^
Δρ_min_ = −0.16 e Å^−3^

### 

Data collection: *APEX2* (Bruker, 2005 ▶); cell refinement: *SAINT* (Bruker, 2001 ▶); data reduction: *SAINT*; program(s) used to solve structure: *SHELXTL* (Sheldrick, 2008 ▶); program(s) used to refine structure: *SHELXTL*; molecular graphics: *SHELXTL*; software used to prepare material for publication: *SHELXTL*.

## Supplementary Material

Crystal structure: contains datablock(s) global, I. DOI: 10.1107/S1600536812047800/cv5366sup1.cif

Structure factors: contains datablock(s) I. DOI: 10.1107/S1600536812047800/cv5366Isup2.hkl

Supplementary material file. DOI: 10.1107/S1600536812047800/cv5366Isup3.cml

Additional supplementary materials:  crystallographic information; 3D view; checkCIF report

## Figures and Tables

**Table 1:** Hydrogen-bond geometry (Å, °) *Cg* is the centroid of the N1/C2–C6 ring.

*D*—H⋯*A*	*D*—H	H⋯*A*	*D*⋯*A*	*D*—H⋯*A*
N2—H2*A*⋯N1^i^	0.896 (17)	2.211 (17)	3.1062 (14)	177.5 (11)
N2—H2*B*⋯*Cg* ^ii^	0.867 (17)	2.674 (16)	3.4875 (12)	163.5 (11)

Symmetry codes: (i) 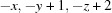; (ii) 
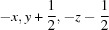
.

**Fig. 1.**
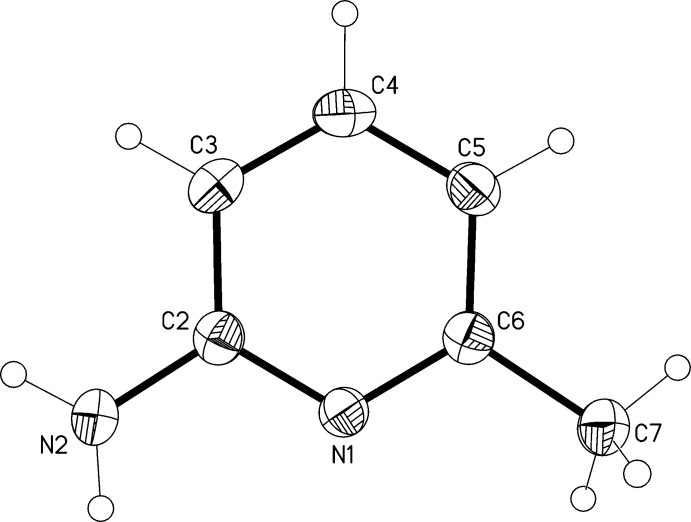
Molecular structure of I. Displacement ellipsoids are shown at the 50% probability level. H atoms are presented as small spheres of arbitrary radius.

**Fig. 2.**
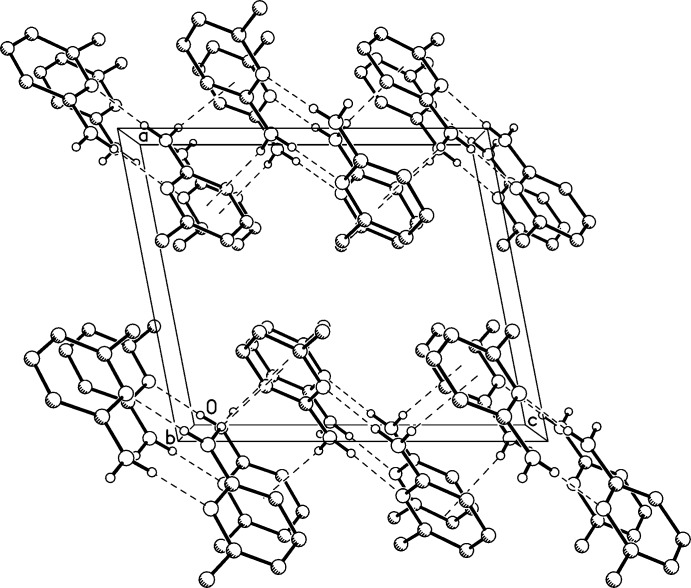
A portion of the crystal packing showing intermolecular N—H···N and N—H···π hydrogen bonds as dashed lines.

## supplementary crystallographic information

### Comment

In supramolecular chemistry, intermolecular non-valent interactions, as a factor responsible for the collective properties of solids, are useful chemical tools to control stability, conformation, and assembly of molecules and thus to design new materials with specific physical and chemical properties (Scheiner, 1997). In particular, the absolute asymmetric synthesis that affords optically active compounds starting from achiral reactants in the absence of any external chiral agents is of significant interest (Jacques *et al.*, 1981). To enable the absolute asymmetric synthesis with a high reliability, it is necessary to predict and obtain chiral crystals through self-assembly of the achiral molecules. Such chiral co-crystals are very important as starting solids for the nonlinear optical materials (Miyata, 1991).

In this paper, we determined the structure of the title compound (I), C_6_H_8_N_2_ (Figure 1), with the purpose to study the strengths and directional propensities of its intermolecular non-bonding interactions and to generate in future the chiral molecular co-crystals on the basis of this compound. The structures of several interesting series with pyridine-2-amino-6-methyl derivatives, including acentric organic salts, have been already reported (Büyükgüngör & Odabaşoǧlu, 2006; Chtioui & Jouini, 2006; Ni *et al.*, 2007; Dai *et al.*, 2011; Waddell *et al.*, 2011).

In the molecule of I, endocyclic angles cover the range 118.43 (9)–122.65 (10)°. The endocyclic angles at the C2 and C6 carbon atoms adjacent to the N1 heteroatom are larger than 120°, and those at the other atoms of the ring are smaller than 120°. All the non-hydrogen atoms lie within the same plane (r.m.s. deviation is 0.007 Å). The N2 atom of the amino group has a slightly pyramidalized configuration (sum of the bond angles is 356°).

In the crystal of I, the pyridine N1 atom serves as the acceptor of the N—H···N hydrogen bond (Table 1) which links two molecules into the centrosymmetric dimer (Figure 2). The intermolecular N—H···π interaction (Table 1) between the amino group and pyridine ring further consolidate the crystal packing, forming the layers parallel to (100) (Figure 2).

### Experimental

The compound I was obtained commercially (Aldrich) as a fine-crystalline powder and purified additionally by filtration. Crystals suitable for the X-ray diffraction study were grown by slow evaporation from chloroform solution.

### Refinement

The hydrogen atoms of the amino group were localized in the difference-Fourier map and refined isotropically. The other hydrogen atoms were placed in the calculated positions with C—H = 0.93 Å (CH-groups) and 0.96 Å (CH_3_-group) and refined in the riding model with fixed isotropic displacement parameters [*U*_iso_(H) = 1.5*U*_eq_(C) for the CH_3_-group and 1.2*U*_eq_(C) for the CH-groups].

### Crystal data

**Table:**

C_6_H_8_N_2_	*F*(000) = 232
*M**_r_* = 108.14	*D*_x_ = 1.219 Mg m^−^^3^
Monoclinic, *P*2_1_/*c*	Mo *K*α radiation, λ = 0.71073 Å
Hall symbol: -P 2ybc	Cell parameters from 2436 reflections
*a* = 9.1006 (11) Å	θ = 2.3–30.0°
*b* = 6.2458 (8) Å	µ = 0.08 mm^−^^1^
*c* = 10.5598 (13) Å	*T* = 296 K
β = 100.952 (2)°	Prism, colourless
*V* = 589.29 (13) Å^3^	0.30 × 0.25 × 0.20 mm
*Z* = 4	

### Data collection

**Table:**

Bruker APEXII CCD diffractometer	1420 independent reflections
Radiation source: fine-focus sealed tube	1196 reflections with *I* > 2σ(*I*)
Graphite monochromator	*R*_int_ = 0.030
φ and ω scans	θ_max_ = 28.0°, θ_min_ = 2.3°
Absorption correction: multi-scan (*SADABS*; Sheldrick, 2003)	*h* = −12→12
*T*_min_ = 0.977, *T*_max_ = 0.985	*k* = −8→8
5852 measured reflections	*l* = −13→13

### Refinement

**Table:**

Refinement on *F*^2^	Primary atom site location: structure-invariant direct methods
Least-squares matrix: full	Secondary atom site location: difference Fourier map
*R*[*F*^2^ > 2σ(*F*^2^)] = 0.043	Hydrogen site location: difference Fourier map
*wR*(*F*^2^) = 0.130	H atoms treated by a mixture of independent and constrained refinement
*S* = 1.00	*w* = 1/[σ^2^(*F*_o_^2^) + (0.082*P*)^2^ + 0.126*P*] where *P* = (*F*_o_^2^ + 2*F*_c_^2^)/3
1420 reflections	(Δ/σ)_max_ < 0.001
82 parameters	Δρ_max_ = 0.32 e Å^−^^3^
0 restraints	Δρ_min_ = −0.16 e Å^−^^3^

### Special details

**Table:**

Geometry. All e.s.d.'s (except the e.s.d. in the dihedral angle between two l.s. planes) are estimated using the full covariance matrix. The cell e.s.d.'s are taken into account individually in the estimation of e.s.d.'s in distances, angles and torsion angles; correlations between e.s.d.'s in cell parameters are only used when they are defined by crystal symmetry. An approximate (isotropic) treatment of cell e.s.d.'s is used for estimating e.s.d.'s involving l.s. planes.
Refinement. Refinement of *F*^2^ against ALL reflections. The weighted *R*-factor *wR* and goodness of fit *S* are based on *F*^2^, conventional *R*-factors *R* are based on *F*, with *F* set to zero for negative *F*^2^. The threshold expression of *F*^2^ > σ(*F*^2^) is used only for calculating *R*-factors(gt) *etc*. and is not relevant to the choice of reflections for refinement. *R*-factors based on *F*^2^ are statistically about twice as large as those based on *F*, and *R*- factors based on ALL data will be even larger.

### Fractional atomic coordinates and isotropic or equivalent isotropic displacement parameters (Å^2^)

**Table:**

	*x*	*y*	*z*	*U*_iso_*/*U*_eq_	
N1	0.17331 (10)	0.50475 (14)	0.92049 (8)	0.0227 (3)	
N2	−0.00315 (12)	0.77129 (17)	0.90952 (10)	0.0311 (3)	
H2A	−0.0502 (18)	0.693 (3)	0.9607 (16)	0.040 (4)*	
H2B	−0.0533 (18)	0.877 (3)	0.8697 (15)	0.038 (4)*	
C2	0.11252 (12)	0.68485 (17)	0.86263 (10)	0.0234 (3)	
C3	0.16561 (13)	0.77898 (18)	0.75860 (11)	0.0271 (3)	
H3	0.1219	0.9029	0.7196	0.033*	
C4	0.28303 (13)	0.68378 (19)	0.71621 (10)	0.0281 (3)	
H4	0.3191	0.7419	0.6470	0.034*	
C5	0.34831 (12)	0.49983 (18)	0.77700 (10)	0.0267 (3)	
H5	0.4291	0.4349	0.7500	0.032*	
C6	0.29036 (12)	0.41577 (17)	0.87838 (10)	0.0234 (3)	
C7	0.35535 (13)	0.21888 (19)	0.94938 (11)	0.0307 (3)	
H7A	0.4172	0.2597	1.0298	0.046*	
H7B	0.4146	0.1429	0.8980	0.046*	
H7C	0.2758	0.1282	0.9656	0.046*	

### Atomic displacement parameters (Å^2^)

**Table:**

	*U*^11^	*U*^22^	*U*^33^	*U*^12^	*U*^13^	*U*^23^
N1	0.0270 (5)	0.0204 (4)	0.0207 (4)	0.0013 (3)	0.0052 (3)	−0.0013 (3)
N2	0.0372 (6)	0.0251 (5)	0.0331 (5)	0.0101 (4)	0.0122 (4)	0.0046 (4)
C2	0.0256 (5)	0.0207 (5)	0.0233 (5)	−0.0009 (4)	0.0029 (4)	−0.0030 (4)
C3	0.0292 (6)	0.0236 (5)	0.0271 (5)	−0.0005 (4)	0.0018 (4)	0.0045 (4)
C4	0.0271 (6)	0.0331 (6)	0.0240 (5)	−0.0056 (4)	0.0047 (4)	0.0045 (4)
C5	0.0243 (5)	0.0315 (6)	0.0251 (5)	0.0014 (4)	0.0065 (4)	−0.0009 (4)
C6	0.0254 (5)	0.0225 (5)	0.0218 (5)	0.0002 (4)	0.0031 (4)	−0.0027 (4)
C7	0.0351 (6)	0.0269 (6)	0.0316 (6)	0.0078 (5)	0.0098 (5)	0.0031 (4)

### Geometric parameters (Å, º)

**Table:**

N1—C2	1.3476 (14)	C4—C5	1.3929 (16)
N1—C6	1.3496 (13)	C4—H4	0.9300
N2—C2	1.3575 (14)	C5—C6	1.3837 (15)
N2—H2A	0.896 (17)	C5—H5	0.9300
N2—H2B	0.867 (17)	C6—C7	1.5019 (15)
C2—C3	1.4099 (15)	C7—H7A	0.9600
C3—C4	1.3702 (16)	C7—H7B	0.9600
C3—H3	0.9300	C7—H7C	0.9600
			
C2—N1—C6	118.43 (9)	C6—C5—C4	118.53 (10)
C2—N2—H2A	119.7 (10)	C6—C5—H5	120.7
C2—N2—H2B	120.1 (10)	C4—C5—H5	120.7
H2A—N2—H2B	116.2 (14)	N1—C6—C5	122.65 (10)
N1—C2—N2	116.52 (10)	N1—C6—C7	115.68 (9)
N1—C2—C3	121.96 (10)	C5—C6—C7	121.67 (10)
N2—C2—C3	121.51 (10)	C6—C7—H7A	109.5
C4—C3—C2	118.53 (10)	C6—C7—H7B	109.5
C4—C3—H3	120.7	H7A—C7—H7B	109.5
C2—C3—H3	120.7	C6—C7—H7C	109.5
C3—C4—C5	119.88 (10)	H7A—C7—H7C	109.5
C3—C4—H4	120.1	H7B—C7—H7C	109.5
C5—C4—H4	120.1		
			
C6—N1—C2—N2	−178.81 (9)	C3—C4—C5—C6	1.01 (17)
C6—N1—C2—C3	1.34 (15)	C2—N1—C6—C5	−1.20 (16)
N1—C2—C3—C4	−0.31 (17)	C2—N1—C6—C7	178.40 (9)
N2—C2—C3—C4	179.84 (10)	C4—C5—C6—N1	0.04 (17)
C2—C3—C4—C5	−0.88 (17)	C4—C5—C6—C7	−179.54 (10)

### Hydrogen-bond geometry (Å, º)

Cg is the centroid of the N1/C2–C6 ring.

**Table:**

*D*—H···*A*	*D*—H	H···*A*	*D*···*A*	*D*—H···*A*
N2—H2*A*···N1^i^	0.896 (17)	2.211 (17)	3.1062 (14)	177.5 (11)
N2—H2*B*···*Cg*^ii^	0.867 (17)	2.674 (16)	3.4875 (12)	163.5 (11)

Symmetry codes: (i) −*x*, −*y*+1, −*z*+2; (ii) −*x*, *y*+1/2, −*z*−1/2.
